# Tussilagone Inhibits Osteoclastogenesis and Periprosthetic Osteolysis by Suppressing the NF-κB and P38 MAPK Signaling Pathways

**DOI:** 10.3389/fphar.2020.00385

**Published:** 2020-04-03

**Authors:** Xuantao Hu, Ziqing Yin, Xia Chen, Guangyao Jiang, Daishui Yang, Ziqin Cao, Shuai Li, Zicheng Liu, Dan Peng, Pengcheng Dou

**Affiliations:** Department of Orthopedics, The Second Xiangya Hospital of Central South University, Changsha, China

**Keywords:** tussilagone, osteoclast, NF-κB, p38, MAPK, aseptic prosthetic loosening, periprosthetic osteolysis

## Abstract

**Background:**

Aseptic prosthetic loosening is one of the main factors causing poor prognosis of limb function after joint replacement and requires troublesome revisional surgery. It is featured by wear particle-induced periprosthetic osteolysis mediated by excessive osteoclasts activated in inflammatory cell context. Some natural compounds show antiosteoclast traits with high cost-efficiency and few side effects. Tussilagone (TUS), which is the main functional extract from *Tussilago farfara* generally used for relieving cough, asthma, and eliminating phlegm in traditional medicine has been proven to appease several RAW264.7-mediated inflammatory diseases *via* suppressing osteoclast-related signaling cascades. However, whether and how TUS can improve aseptic prosthetic loosening *via* modulating osteoclast-mediated bone resorption still needs to be answered.

**Methods:**

We established a murine calvarial osteolysis model to detect the preventative effect of TUS on osteolysis *in vivo*. Micro-CT scanning and histomorphometric analysis were used to determine the variation of bone resorption and osteoclastogenesis. The anti–osteoclast-differentiation and anti–bone-resorption bioactivities of TUS *in vitro* were investigated using bone slice resorption pit evaluation, and interference caused by cytotoxicity of TUS was excluded according to the CCK-8 assay results. Quantitative polymerase chain reaction (qPCR) analysis was applied to prove the decreased expression of osteoclast-specific genes after TUS treatment. The inhibitory effect of TUS on NF-κB and p38 MAPK signaling pathways was testified by Western blot and NF-κB-linked luciferase reporter gene assay.

**Results:**

TUS better protected bones against osteolysis in murine calvarial osteolysis model with reduced osteoclasts than those in the control group. *In vitro* studies also showed that TUS exerted antiosteoclastogenesis and anti–bone-resorption effects in both bone marrow macrophages (BMMs) and RAW264.7 cells, as evidenced by the decline of osteoclast-specific genes according to qPCR. Western blotting revealed that TUS treatment inhibited IκBα degradation and p38 phosphorylation.

**Conclusions:**

Collectively, our studies proved for the first time that TUS inhibits osteoclastogenesis by suppressing the NF-κB and p38 MAPK signaling pathways, therefore serving as a potential natural compound to treat periprosthetic osteolysis-induced aseptic prosthetic loosening.

## Introduction

The skeletal system bears body weight and supports body shape with its rigidity, while the normal metabolism of bone tissue relies on a dynamic balance between bone generation and resorption mediated by the osteoblast and osteoclast respectively (Ouyang et al., 2019; Zhu et al., 2019). Contrarily, the pathological generation of osteoclast has been discovered as the “culprit” of various orthopedic disorders manifesting as bone loss or osteolysis, like aseptic prosthetic loosening of artificial joint, postmenopausal osteoporosis, and bone metastasis of some cancers (Crotti et al., 2004; Rachner et al., 2011; Maurizi and Rucci, 2018), which produce poor prognosis and demand healthcare. Especially, inflammatory cytokine- and chemokine-induced osteoclast generation seems to be a major cause of periprosthetic osteolysis (Goodman and Ma, 2010). Given the situation, therapeutic treatment against osteoclast over-production has great significance in the clinical management of aseptic prosthetic loosening.

Osteoclast is a highly-differentiated multinuclear giant cell comprising fused monocyte–macrophage progenitors originating from hematopoietic lineage (Ono and Nakashima, 2018), like BMMs and RAW264.7. Many studies have unraveled the molecular mechanism of osteoclastogenesis and several canonical pathways have been clarified. As a leading pro-osteoclastogenesis factor, the receptor activator of nuclear factor κB ligand (RANKL) initiates a primary signal by binding to the RANK located in the membrane to activate TNF receptor-associated factor 6 (TRAF 6) (Asagiri and Takayanagi, 2007). TRAF 6 plays a pivotal role in the promotion of PI3K-Akt, NF-κB, and MAPK signaling pathways (including JNK, ERK, and p38 pathways) by upregulating the Src, IκB kinase (IKK) complex, and MEK/MKK respectively (Cao et al., 2010; Ping et al., 2017; Wang et al., 2018). CaMK-CREB and Jak-STAT pathways also participate in regulating the transcription of osteoclastic bone resorption related genes (Sato et al., 2006; Farr et al., 2017). Activation of these cascades are responsible for the increase in expression of specific genes, like Cathepsin K, tartrate-resistant acid phosphatase (TRAP), and c-Fos that are involved in cytoskeletal rearrangement during macrophage fusion (Helming and Gordon, 2009; Liu et al., 2014), which regulates the bio-function of osteoclasts. Hence, agents targeting canonical pathways like NF-κB and MAPK may be of antiosteoclastogenetic property for the management of aseptic prosthetic loosening.

Tussilagone (TUS) is a sesquiterpenoid isolated from the flower of *Tussilago farfara* or some other species in the genus *Tussilago*. Previous studies have suggested that this natural compound exerts therapeutic effects in inflammatory pulmonary diseases (Choi et al., 2018), inflammatory intestinal diseases (IBD) (Cheon et al., 2018), ischemic stroke (Hwang et al., 2018), colon cancer (Li et al., 2014), obesity and type 2 diabetes (Park et al., 2008), demonstrating diverse effect in regulating pathophysiological changes like anti-inflammation (Hwangbo et al., 2009; Lee et al., 2016; Kim et al., 2017b), antioxidation (Park et al., 2014; Qin et al., 2014), antitumor (Li et al., 2014). Especially, TUS was found to attenuate lipopolysaccharide (LPS)-induced inflammatory mediator production in RAW264.7 cells *via* NF-κB and (or) MAPK signaling cascades (Hwangbo et al., 2009; Lee et al., 2016; Kim et al., 2017b), therefore it may be a possible drug candidate for bone protection by suppressing osteoclast formation and activation. However, except for the above-mentioned effects towards osteoclast progenitors and other cell lines, little is known about whether and how TUS suppresses RANKL-induced osteoclast differentiation and improves periprosthetic osteolysis-induced aseptic prosthetic loosening *in vivo*. Therefore, we designed a study to investigate the therapeutic benefits of TUS for osteoclast and illuminate the underlying molecular mechanism, thus complementing the theory and suggesting greater value of TUS in the treatment of osteolytic diseases.

## Materials and Methods

### Reagents and Cell Preparation

TUS (purity >98%, **Figure 3A**) was purchased from Dalian Meilun Biotechnology (Liaoning, China) and dissolved in alpha modification of minimum essential medium (α-MEM; Gibco-BRL; Gaithersburg, MD, USA) to prepare a 0.2 M solution stored at 4°C. The fetal bovine serum (FBS), penicillin/streptomycin, soluble mouse recombinant M-CSF, and RANKL were acquired from R&D Systems (Minneapolis, MN, USA). The cell counting kit (CCK-8) was obtained from Dojindo Molecular Technology (Japan) The DMSO and TRAP staining kit was obtained from Sigma-Aldrich (St. Louis, MO, USA).

RAW264.7 cells were purchased from American Type Culture Collection (Rockville, MD, USA) and incubated in α-MEM supplemented with 10% FBS and 1% penicillin/streptomycin, namely complete α-MEM. C57BL/6 mice (4- to 6-week-old) were acquired from Shanghai Laboratory Animal Company (SLACCAS, Shanghai, China). Primary BMMs were separated from the whole bone marrow of murine femurs and tibias and cultured in complete α-MEM supplemented with 30 ng/ml M-CSF. All cells used in this study were incubated at constant high humidity, 37°C, and 5% CO_2_ atmosphere (Zhang et al., 2018). Nonadherent cells were removed before each passage.

### Cell Viability

The cytotoxic effects of TUS on RAW264.7 and BMM cells were assessed using a CCK-8 assay according to the instruction book. RAW264.7 or BMM cells (3×10^3^ cells per well) were plated in 96-well plates till adhesion overnight. Cells were then treated with diverse concentrations of TUS (0, 0.39, 0.78, 1.56, 3.13, 6.25, 12.5, 25, 50, or 100 µM) for 48 or 96 h. A 10-μl volume of CCK-8 buffer was added to each well and incubated at 37°C for an additional 1 h. Then, the optical density (OD) was detected at 450-nm wavelength (650-nm reference) on an ELX800 absorbance microplate reader (Bio-Tek, USA). Cell viability was calculated relative to control using the following formula: (experimental group OD - zeroing OD)/(control group OD - zeroing OD) (Zhou et al., 2017).

### Osteoclast Formation Assay

To investigate the effect of TUS on osteoclast formation *in vitro*, RAW264.7 or BMM cells (2.5×10^3^ cells per well) were seeded in 96-well plates and then cultured overnight. After confirming the healthy condition of the cells, they were cultured with complete α-MEM with 50 ng/ml RANKL (30 ng/ml M-CSF for BMMs solely) and different noncytotoxic concentrations of TUS (0, 6.25, 12.5, and 25 µM), until osteoclast formation was clearly observed 5 d later. The osteoclasts were fixed with 4% paraformaldehyde for 20 min and then stained for TRAP. TRAP-positive cells with more than five nuclei were counted as osteoclasts.

### Bone Resorption Pit Evaluation

RAW264.7 cells (3×10^3^ cells per well) were plated on the surface of sterile bovine bone slices in 96-well plates with complete α-MEM containing 50 ng/ml RANKL (30 ng/ml M-CSF for BMMs solely) and different concentrations of TUS (0, 6.25, 12.5, 25 µM). After confirming osteoclast formation, the cells were treated with complete culture medium supplemented with 30 ng/ml M-CSF, 50 ng/ml RANKL, and different TUS concentrations for another 48 h. Finally, bone resorption effect on the bovine bone slices was evaluated using a scanning electron microscope (SEM; FEI Quanta 250). Area of the bone resorption relative to control were quantified by Image J software.

### RNA Extraction and qPCR Analysis

Quantitative PCR was applied to measure the expression of osteoclast-specific genes. BMMs (1×10^5^ cells per well) were seeded in 24-well plates with complete α-MEM, 50 ng/ml RANKL and 30 ng/ml M-CSF. The cells were administered with different concentrations of TUS (0, 6.25, 12.5, or 25 µM). Then, RNA was extracted with the Qiagen RNeasy Mini kit (Qiagen; Valencia, CA, USA) according to the instructions and cDNA was synthesized from 1 mg total RNA using a reverse transcriptase kit (TaKaRa Biotechnology; Otsu, Japan). The SYBR Premix Ex Tag kit (TaKaRa Biotechnology) and an ABI 7500 Sequencing Detection System (Applied Biosystems; Foster City, CA, USA) was used in qPCR. PCR conditions were 40 cycles of denaturation at 95°C for 5 s and amplification at 60°C for 34 s (Zhang et al., 2018). The reactions were conducted in triplicate. Measured results were normalized to the expression of GAPDH. The mice primer sequence set is listed in **Table 1**.

**Table 1:** Murine primer sequence set for quantitative polymerase chain reaction (qPCR).

Gene	Forward primer	Reverse primer
Calcr	5′-CGGACTTTGACACAGCAGAA-3′	5′-AGCAGCAATCGACAAGGAGT-3′
Cathepsin K	5′-CTTCCAATACGTGCAGCAGA-3′	5′-TCTTCAGGGCTTTCTCGTTC-3′
c-Fos	5′-CCAGTCAAGAGCATCAGCAA-3′	5′-AAGTAGTGCAGCCCGGAGTA-3′
Dc-stamp	5'-AAAACCCTTGGGCTGTTCTT-3'	5'-AATCATGGACGACTCCTTGG-3'
Nfatc1	5′-CCGTTGCTTCCAGAAAATAACA-3′	5′-TGTGGGATGTGAACTCGGAA-3′
Traf6	5′-AAACCACGAAGAGGTCATGG-3′	5′-GCGGGTAGAGACTTCACAGC-3′
Gapdh	5′-ACCCAGAAGACTGTGGATGG-3′	5′-CACATTGGGGGTAGGAACAC-3′

### NF-κB Luciferase Reporter Gene Assay

To examine whether TUS affected NF-κB gene expression, RAW264.7 cells were stably transfected with a p-NF-κB-TA-Luc luciferase reporter construct. Concisely, 1×10^5^ cells per well were plated in a 24-well plate for 24 h. Then, they were pretreated with different concentrations of TUS (0, 6.25, 12.5, 25 µM) for 1 h, prior to incubation with 50 ng/ml RANKL for another 8 h. Cells were then lysed and luciferase activity was measured using the Promega Luciferase Assay System according to the instruction book (Liu et al., 2014).

### Western Blot Analysis

RAW264.7 cells (5×10^5^ cells per well) were seeded in 6-well plates. When the cells were confluent, they were pretreated with or without 25 µM TUS for 4 h and then incubated with 50 ng/ml RANKL for 0, 5, 10, 20, 30, or 60 min. Total protein was extracted from cultured cells using the radioimmunoprecipitation assay (RIPA) lysis buffer (Well Biology, Changsha, China) with protease inhibitor cocktail. Next, the lysates were centrifuged at 12,000× *g* for 15 min, and the supernatants that contained the proteins were collected. Protein concentrations were measured with bicinchoninic acid assay (Well Biology).

Each cell lysate (30 mg) was resolved on 10% sodium dodecyl sulfate polyacrylamide gel. The products were transferred to polyvinylidene difluoride membranes (Millipore; Bedford, MA, USA), which were then blocked with 5% skimmed milk powder in TBS-Tween (TBS: 0.05 M Tris, 0.15 M NaCl, pH 7.5, and 0.2% Tween-20) for 1 h and incubated with primary antibodies overnight at 4°C (Liu et al., 2014). Membranes were then rinsed with TBS-Tween and incubated with the corresponding secondary antibodies conjugated with IRDye 800CW (molecular weight 1,162 Da) for 2 h. Bands were detected through the Odyssey infrared imaging system (LI-COR Bioscience; Lincoln, NE, USA). Quantitative analysis of band intensity was performed by ImageJ software (National Institutes of Health; MD, USA).

### Ti Particle-Induced Calvarial Osteolysis Model

This study was carried out in accordance with the recommendations of guiding principles of Animal Care Committee of Central South University. The protocol was also approved by the Animal Care Committee of Central South University.

A murine calvarial osteolysis model was established to determine the preventative effects of TUS on osteolysis *in vivo*. In short, 16 healthy 8-week-old C57BL/6 mice (weight: 21.47 ± 1.22 g) bred in specific pathogen-free (SPF) plastic-isolator cages were assigned randomly into three groups: sham phosphate buffer solution (PBS) control (sham), Ti particles with PBS (vehicle), and Ti particles with 10 mg/kg/d TUS (TUS group). To remove endotoxins adherent on Ti particles, commercial pure Ti particles were sterilized by baking at 180°C for 6 h, followed by 70% ethanol treatment for 2 d. The mice were anesthetized by ketamine (80 mg/kg bw; Hengrui Medicine Co Ltd.; Jiangsu, China) and xylazine (10 mg/kg bw; Baide Biomedicine; Qingdao, China) administered intraperitoneally and the cranial periosteum was separated from the calvarium by sharp dissection. Then, 30 mg Ti particles were implanted under the periosteum at the middle suture of the calvarium (Liu et al., 2014). Mice in TUS group were injected intraperitoneally with 10 mg/kg/d TUS for 8 weeks. Mice in the sham and vehicle groups received PBS daily. At the end of the experiment, the mice were euthanized using pentobarbital sodium (100 mg/kg bw; Hengrui Medicine Co Ltd.; Jiangsu, China) administered intraperitoneally before cervical dislocation and the calvaria were excised and fixed in 4% paraformaldehyde for micro-computed tomography (CT) analysis.

### Micro-CT Scanning

The fixed calvaria were observed using a high-resolution micro-CT system (μCT50; Scanco; Zurich; Switzerland). The scanning protocol was set at an isometric resolution at 8.3 mm and X-ray energy settings at 80 kV and 80 mA. After reconstruction, a square region of interest around the midline suture was selected for further qualitative and quantitative analysis. Bone volume against tissue volume (BV/TV), the number of pores, and percentage of total porosity of each sample were analyzed.

### Histomorphometric Analysis

After micro-CT scanning, the calvaria samples were decalcified in 10% ethylene diamine tetraacetic acid (EDTA) for 3 weeks and embedded in paraffin. Histological sections were preconditioned for TRAP and hematoxylin and eosin (H&E) staining. The specimens were then examined and photographed under a high quality microscope. The number of TRAP-positive multinucleated osteoclasts was counted in each sample.

### Statistical Analysis

All experiments were performed at least thrice. The data are expressed as means ± SD. Results were analyzed using the Student's *t* test using the SPSS 13.0 software (SPSS Inc.; USA). P < 0.05 indicated a significant difference between the results of different groups.

## Results

### TUS Alleviates Ti Particle-Induced Osteolysis in Mice

**Figure 1:**
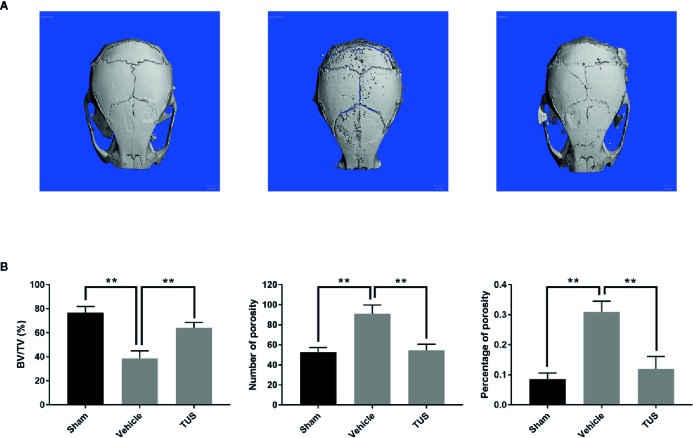
TUS inhibited Ti particle-induced murine calvarium osteolysis. **(A)** Representative three-dimensional reconstructed images of calvarium of micro-computed tomography (micro-CT) from each group. **(B)** Bone volume against tissue volume (BV/TV), number of pores, and the percentage of total porosity of each sample was measured. (**: P < 0.01 versus control group).

TUS was previously proved to alleviate LPS-stimulated inflammatory reaction in RAW264.7 and cecal ligation and puncture (CLP)-induced septic murine model by downregulating NF-κB and MAPK pathways (Kim et al., 2017b). However, osteoclast over-activation by aseptic inflammation is also a main factor of osteoclast-dominated osteolytic diseases like aseptic prosthetic loosening. Thus, we investigated the anti–bone-resorption bioactivity of TUS in the developed Ti particle-induced murine calvarial osteolysis model. Micro-CT with three-dimensional reconstruction demonstrated intensive bone resorption in the vehicle group than that in the sham group, while particle-induced osteolysis was reduced in the TUS treatment group (**Figure 1A**). The measurements of bone volume/total volume (BV/TV), number of porosity, and the percentage of total porosity in the region of interest proved evident Ti particle-induced osteolysis in the vehicle group. In the 10 mg/kg/d TUS treatment group, osteolysis was significantly attenuated than that in the vehicle group (**Figure 1B**).

**Figure 2:**
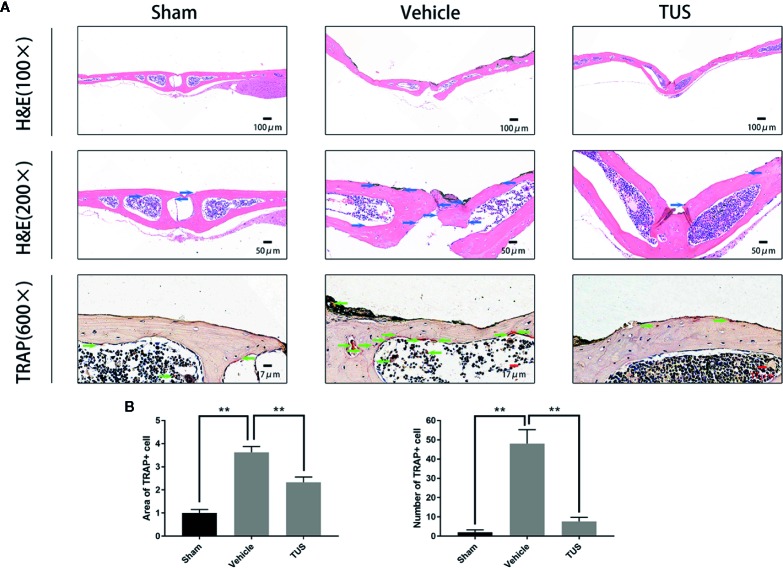
**(A)** Inhibition of Ti particle-induced murine calvarial osteolysis by Tussilagone (TUS) as assessed using immunohistochemical staining analysis. Hematoxylin and eosin (HE) and tartrate-resistant acid phosphatase (TRAP) staining were performed on at least three sections per group. **(B)** The area and the number of TRAP positive cells of each sample was measured. (**: P < 0.01 versus control group).

Histomorphometric analysis further confirmed TUS-mediated anti–bone-resorption. The presence of Ti particles induced the inflammatory infiltration of lymphocytes and macrophages, as well as multinucleated osteoclasts at the injection site. TRAP staining revealed that multiple osteoclasts lined along the eroded bone surface in Ti group. Corroboratively, the roughness and area of erosion surface reduced in the TUS treatment groups and the number of TRAP-positive osteoclast decreased (**Figure 2**).

### TUS Suppresses Osteoclastogenesis and Bone Resorption Function at Noncytotoxic Concentration *in Vitro*

**Figure 3:**
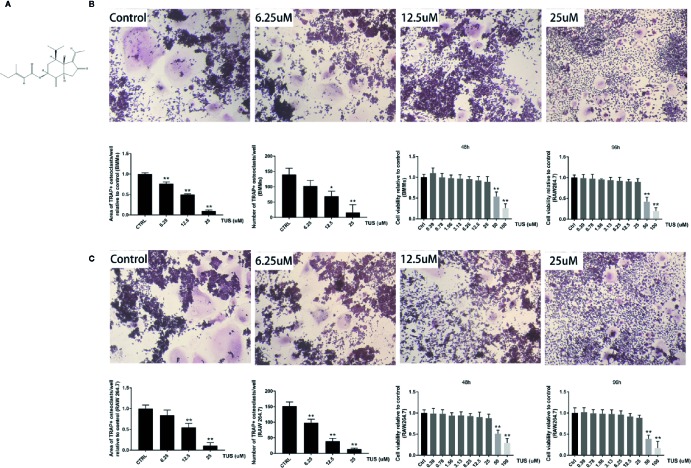
Tussilagone (TUS) suppressed receptor activator of nuclear factor κB ligand (RANKL)–mediated osteoclastogenesis in a dose-dependent manner without cytotoxicity in the RAW264.7 cell line. **(A)** The structural formula of TUS. The microscopic images of RANKL-induced osteoclastogenesis, the area and number of tartrate-resistant acid phosphatase (TRAP)–positive osteoclasts after TUS treatments in **(B)** BMM and **(C)** RAW264.7 cells relative to that in the control group. Cell viability was determined in both types of preosteoclasts at 48 or 96 h respectively. (*: P < 0.05; **: P < 0.01 versus control group).

After confirming the antiosteolysis effect of TUS *in vivo*, the influence of various TUS concentrations on osteoclast differentiation at the cellular level was observed. Numerous TRAP-positive mature multinucleated giant cells derived from RAW264.7 or BMM cells in the control group were clearly recognized. However, the size and number of TRAP-positive mature multinucleated giant cells decreased as TUS concentration gradually increased, which indicated that TUS inhibited osteoclast formation dose-dependently (**Figures 3B, C**).

**Figure 4:**
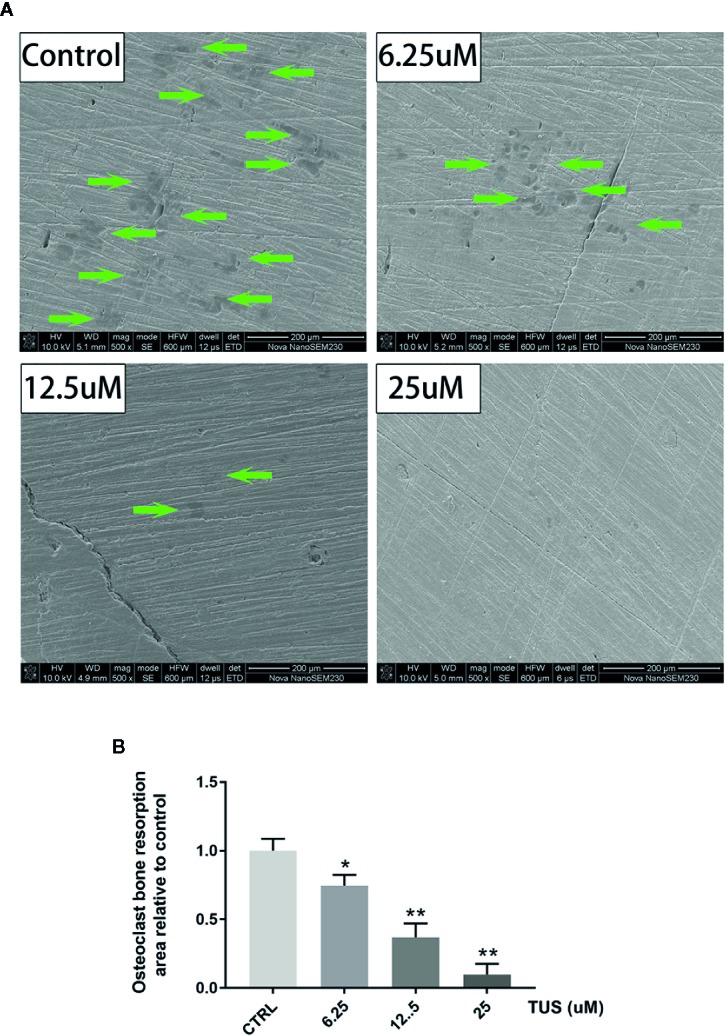
Tussilagone (TUS) treatment dose-dependently mitigated the bone resorption induced by osteoclast at noncytotoxic concentration. **(A)** Scanning electron microscope images of bone resorption pits of all groups. **(B)** Area of the bone resorption relative to control as quantified by ImageJ software. (*: P < 0.05; **: P < 0.01 versus control group).

Furthermore, we hypothesized that TUS downregulates bone resorption function of osteoclasts. SEM visualization demonstrated that TUS groups presented less, smaller, and shallower resorption pits relative to control group (**Figure 4**).

To diminish the possible influence of cytotoxicity, we determined the cytotoxic concentration threshold of TUS using a CCK-8 assay. We discovered that pretreatment with 25 µM TUS or less had no suppressive effect in BMM or RAW264.7 cells (**Figures 3B, C**). Collectively, our data suggest that TUS mitigates osteoclast bone resorption *in vitro*.

### TUS Inhibits Expression of Osteoclast-Specific Genes

**Figure 5:**
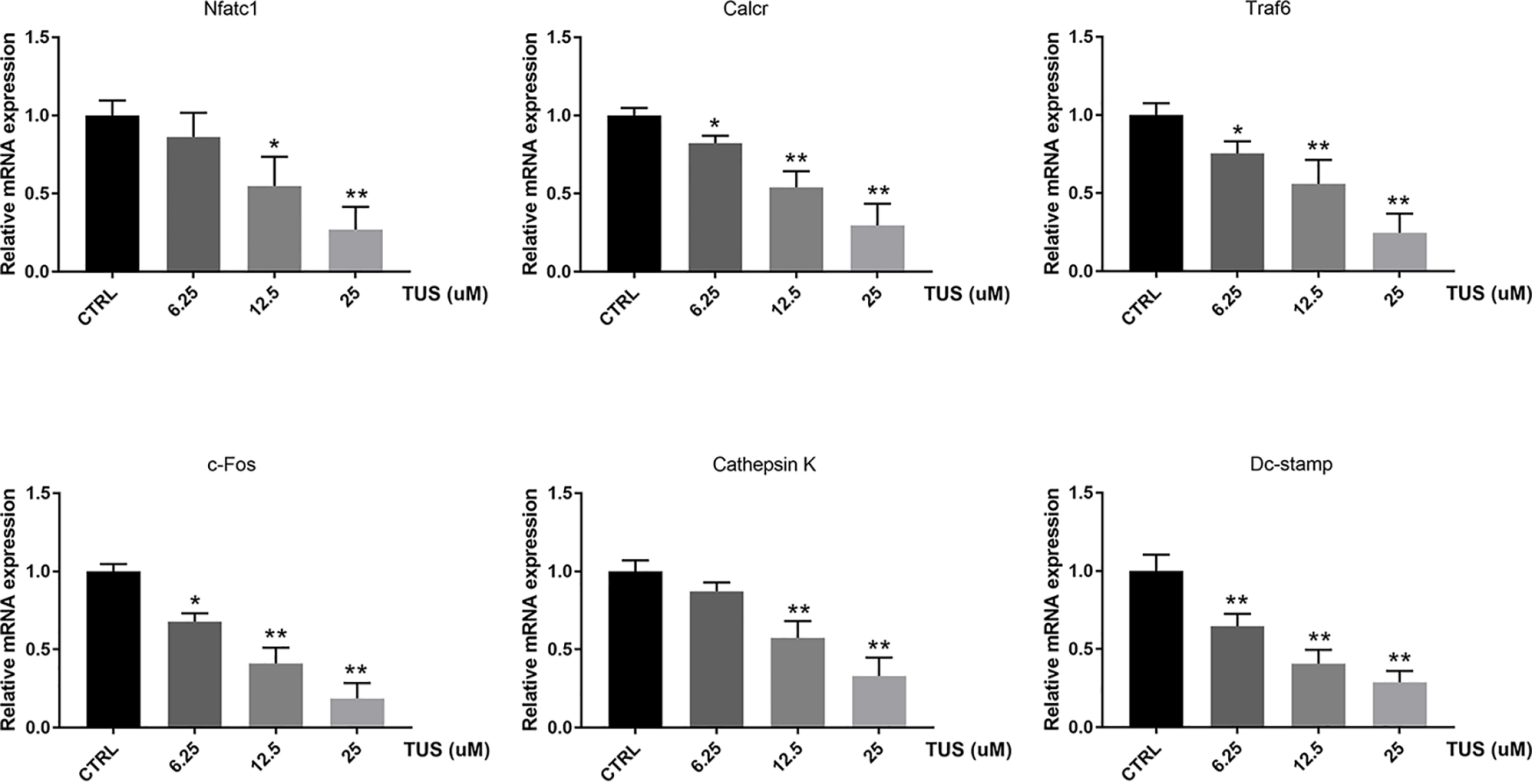
Tussilagone (TUS) hindered transcription of osteoclast-specific genes, including Nfatc1, Calcr, Traf6, c-Fos, Dc-stamp, and Cathepsin K. Measured results were normalized to the expression of Gapdh. (*: P < 0.05; **: P < 0.01 versus control group).

Since osteoclast differentiation is accompanied with RANKL-induced excessive expression of particular genes, we tried to map out their expression alternation after TUS preconditioning using qPCR. We found that mRNA expression of osteoclast-specific genes, including Nfatc1, Calcr, Traf6, c-Fos, Dc-stamp, and Cathepsin K culminated in response to RANKL stimulation in the control group. However, TUS hindered the transcription of these genes dose-dependently (**Figure 5**). These data prove that TUS suppresses osteoclastogenesis by attenuating osteoclast-specific gene expression *in vitro*.

### TUS Hinders RANKL-Induced Activation of NF-κB and P38 Signaling Pathways

**Figure 6:**
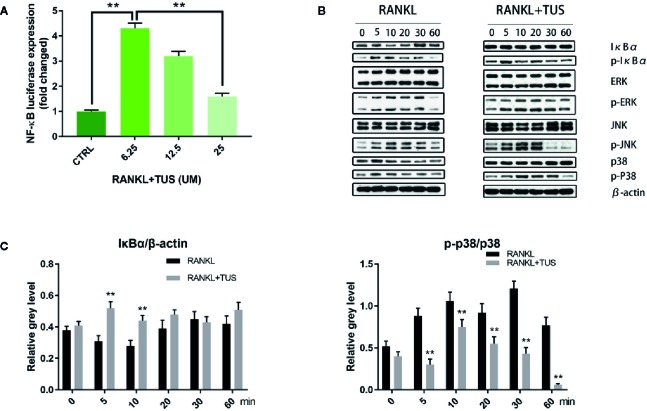
**(A)** Luciferase activity of RAW264.7 cells stably transfected with an NF-κB luciferase reporter construct. **(B)** Tussilagone (TUS) inhibited receptor activator of nuclear factor κB ligand (RANKL)–induced NF-κB and p38-mediated MAPK signaling pathway activation as shown by Western blot of RAW264.7 cell lysates with specific antibodies against p38, p-p38, IκBα, p-IκBα, ERK, p-ERK, JNK, p-JNK, and β-actin. **(C)** Average ratio of IκBα relative to β-actin, p-p38 relative to p38. All experiments were performed at least thrice. (**: P < 0.01 versus control group).

As mentioned before, NF-κB and MAPK are two major pathways in the osteoclastogenesis signaling cascade, so we attempted to elucidate the mechanism underlying the antiosteoclastogenesis effect of TUS by detecting the signaling molecules in NF-κB and MAPK pathways using Western blot. The upstream RANKL-induced phosphorylation of IKK complex, as mentioned before, activates the following signaling cascades of NF-κB and MAPK pathways. IκBα, the inhibitory unit used to bind with NF-κB, disassociates from the location and degrades after being phosphorylated, which leads to the nuclear translocation of subunit p65 and following signaling molecules. The analysis of bands shown that the quantity of IκBα at 5 and 10 min was significantly impaired by TUS treatment (**Figures 6B, C**). Moreover, the suppressive effect of TUS in the NF-κB signaling pathway was further supported by the analysis of NF-κB luciferase reporter gene assay (**Figure 6A**). TUS administration decreased the p-p38 level excessively for the entire period (**Figures 6B, C**).

Apart from this, no obvious alteration was detected in the expression of JNK/P-JNK and ERK/p-ERK with or without TUS treatment.

Collectively, the consequences indicated that TUS targets IκBα degradation and p38 phosphorylation, therefore inhibiting RANKL-mediated NF-κB and MAPK signaling activation.

## Discussion

Periprosthetic osteolysis caused aseptic prosthetic loosening has been considered as the main factor for failure in joint replacement surgery. The limited surgical treatment choices for periprosthetic osteolysis such as complete or partial revision with bone grafting of joint replacement are far from being satisfactory because of recurrence of osteolysis (Adelani et al., 2014). The development of orthopedic material, such as highly cross-linked polyethylene (stabilized with vitamin E or not) for acetabular lining, has an uncertain effect with regard to controlling osteolysis in comparison to ultra-high-molecular-weight polyethylene (Endo et al., 2002; Boyer et al., 2018).

The benefits of existing drugs for bone protection seem to be promising but have considerable adverse effects. Bisphosphonates function by inhibiting the expression of inflammatory mediators and impair osteoclast activity (Iwase et al., 2002), but complications including osteonecrosis of the jaw, hypocalcemia, and renal injury cause latent risks to patients (Peter et al., 2004; Cheng et al., 2018a; Edwards et al., 2008). Denosumab, a humanized monoclonal antibody, specifically combines with RANKL and downregulates RANKL/RANK signaling (Lacey et al., 2012) and shares similar adverse effects with bisphosphonates, like osteonecrosis of the jaw, hypocalcemia, etc. (Iqbal et al., 2010; Chen and Smerdely, 2017; Otto et al., 2018). TNF-α antagonists like Infliximab hinder osteoclastogenesis *via* decreasing, theoretically or actually, RANKL, TNF-α, and M-CSF production (Kitaura et al., 2005; Kim et al., 2017a), while skin irritation like psoriasis and eczema are observed. Hence, exploitation of alternatives for antiosteoclastogenesis drug with high cost-effectiveness and less adverse effects are still of great significance.

Till date, many natural extracts have gained ground in the management of periprosthetic osteolysis (Xiao et al., 2015; Zhao et al., 2016; Meng et al., 2018) due to identical biological safety testified in the longevity of traditional Chinese medicine and affordable prices. *T. farfara* is one of the most commonly used herbal medicines in East Asian and European traditional medicine for treating cough or asthma. TUS, the main component of *T. farfara*, was described to have anti–platelet-aggregation and cardiovascular-respiratory stimulating effects in previous studies (Hwang et al., 1987; Li and Wang, 1988). In recent years, several studies have proven beneficial in the treatment of diseases such as diabetic obesity (Park et al., 2008), cancers (Li et al., 2014), and various inflammatory diseases (Lee et al., 2016; Kim et al., 2017b; Cheon et al., 2018; Choi et al., 2018; Hwang et al., 2018). Additionally, some scholars have studied the metabolic patterns and parameters of TUS and set a foundation for further systematic research in patients (Liu et al., 2008; Zhang et al., 2015; Cheng et al., 2018b). In terms of molecular mechanism, many studies have indicated that TUS suppresses exogenous-agent-stimulated inflammation by downregulating NF-κB signaling in different samples including human colonic tissues, airway epithelial cells, and dendritic and microglial cells (Park et al., 2014; Cheon et al., 2018; Choi et al., 2018; Hwang et al., 2018). Some scholars have reported that TUS may repress the MAPK signaling (including JNK, ERK, and p38 pathways) and then decrease production of inflammatory mediators in different cell lines and animal models (Kim et al., 2017b; Hwang et al., 2018). Moreover, TUS was proven to regulate cancer cell proliferation, angiogenesis, and inflammation *via* Wnt, VEGFR2, and Irf and heme oxygenase-1 (HO-1) pathways, respectively (Li et al., 2014; Park et al., 2014; Li et al., 2019). However, it is not yet understood whether TUS suppresses osteoclastogenesis similarly and it is also not known how it works exactly. Therefore, we designed this study to attempt to elucidate the possible mechanism.

In our work, TUS significantly attenuates multiple index according to micro-CT analysis of bone loss in Ti particle-induced murine calvarial osteolysis model. Immunohistochemical staining analysis shows an obvious drop in the number of osteoclasts in gross and microscopic specimens after TUS pretreatment than that in the control. Then, osteoclast differentiation and bone slice resorption assays indicate not only reduction of number, but also the aggregate biological function caused by TUS at nontoxic concentration dose-dependently, which verify its antiosteoclast characteristic. qPCR results showing that the expression of osteoclast-specific genes including Nfatc1, Calcr, Traf6, c-Fos, Dc-stamp, and Cathepsin K decreases in TUS treatment group also validates this. Finally, Western blot results also indicated that TUS inhibits RANKL-induced osteoclast formation by downregulating the NF-κB and p38/MAPK signaling pathways.

**Figure 7:**
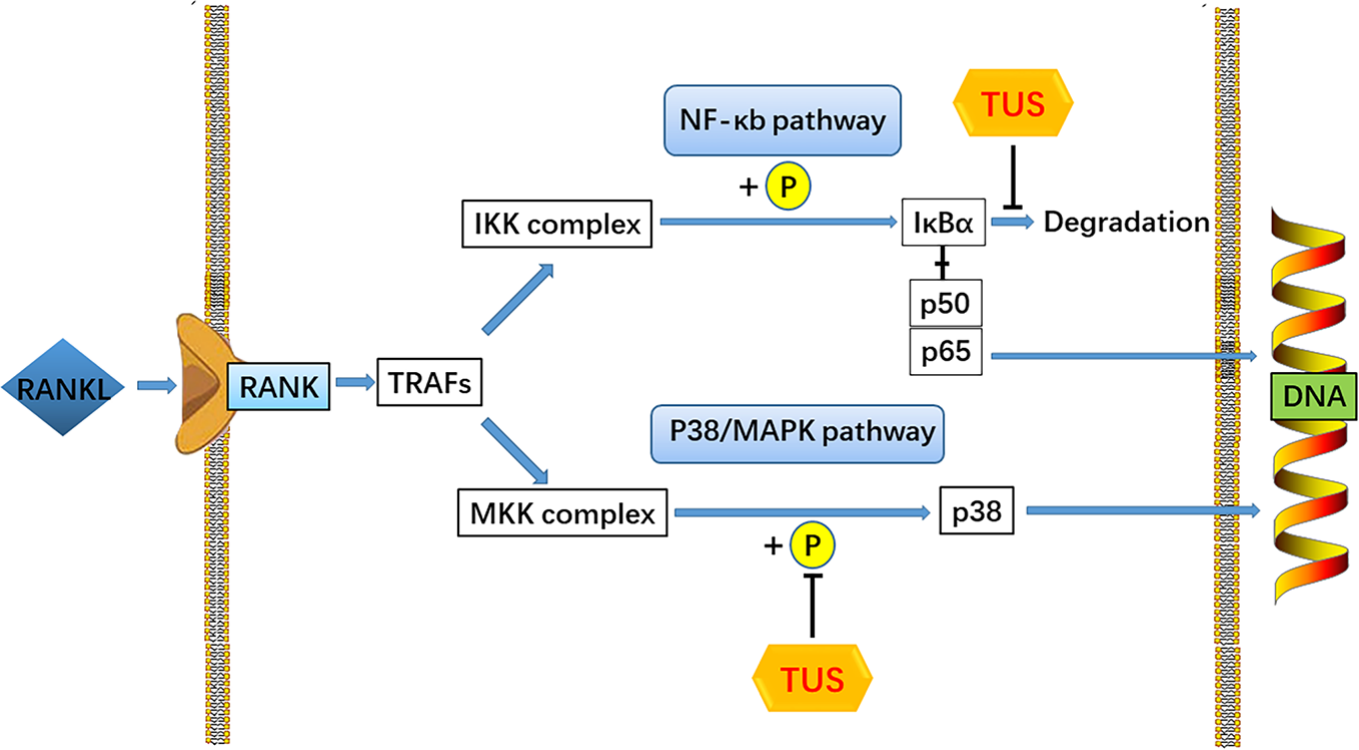
A pattern diagram of Tussilagone (TUS) in downregulating osteoclastogenesis. By targeting IκBα degradation and p38 phosphorylation, TUS suppressed receptor activator of nuclear factor κB ligand (RANKL)–induced expression of osteoclast-specific genes to inhibit osteoclast formation and functions *in vivo* and *in vitro*.

RANKL, one of the key cytokines in monocyte–macrophage lineage, is indispensable for the initiation of osteoclastogenesis and maturation of osteoclasts *in vivo* and *in vitro* (Yin et al., 2019). The specific combination between RANKL and cytomembrane-docking RANK triggers the binding of TRAFs to the cytoplasmic domain of RANK, consequently producing the preliminary signal for following cascades (Boyle et al., 2003). Then, such RANKL/RANK/TRAF complex recruits TGF-β-activated kinase 1 (TAK1), together with the TAK1 binding protein 2 (Boyle et al., 2003; Zhu et al., 2019). TAK1 activates the phosphorylation of IKK complex and sequentially induces the cleavage of NF-κB from the inhibitive protein IκBα (Futosi et al., 2013), which cause the degradation of IκBα and the nuclear translocation of p65 from NF-κB (Ouyang et al., 2019). Moreover, the phosphorylation of p38 following TAK1 activation also plays a role in the signaling induced by RANK-ANKL combination. Taken together, both NF-κB and p38 signaling could trigger sufficient expression of downstream osteoclast-specific genes like Cathepsin K, TRAP, CTR, β3 integrin, and c-Fos, leading to an eventual decrease in bone resorption function (Teitelbaum, 2000). In this study, we adopted the Ti particle-induced murine calvarial osteolysis model to explore the bioactivities of TUS *in vivo*. Significant osteolysis was seen in the vehicle group than that in the control whereas a much more appeased situation in TUS group. Western blot analysis indicates the degradation of IκBα and phosphorylation of p38 apparently diminishes in TUS-treated group relative to that in the control group. To sum up, we conclude that TUS suppresses RANKL-induced osteoclastogenesis by blocking NF-κB and p38-mediated MAPK signaling without affecting JNK and ERK pathways (**Figure 7**) dose-dependently, which is verified in animal, cellular, and molecular levels.

However, there were several limitations in our research.

First, the mice used in this study, has limited cancellous bone, body volume and short lifespan, thus failed to simulate the coexistence of cancellous and cortex bone, decades of wearing period causing long-term inflammatory bio-scene in human body (Gibon et al., 2017), and hardly provided sufficient *in vivo* space and exercise load for implants in studies of their biomechanical properties. Moreover, we made an investigation on the local histology affected by TUS treatment in Ti particle-induced calvarial osteolysis animal model and no significant body weight loss, death or any adverse effect is observed in either control or TUS group in the animal experiment. However, the systematic side effect of TUS was not taken into consideration. Therefore, further investigations with more detailed parameters concerning bio-compatibility and tissue specificity *in vivo* are needed.

Second, Ti particle-induced calvarial osteolysis model, as we used, under-represents the wear-debris-induced osteolysis model used in the emulation of aseptic prosthetic loosening pathogenesis. Different types of wear particles found in the bone-graft interface of patients with different implant materials such as Ti (Eger et al., 2018), cobalt-chromium-molybdenum (CoCrMo) (Wang et al., 2015), ceramics (Gibon et al., 2017), tricalcium phosphate (Lv et al., 2016), polyether-ether-ketone (Du et al., 2018), highly cross-linked polyethylene, and ultra-high-molecular-weight polyethylene (Ormsby et al., 2019) demonstrate various traits in osteolysis. In addition, the limitations that 30 mg Ti particles was locally administrated at one time to produce calvarial osteolysis model was discussed by Liu et al. (Liu et al., 2014) in one of his study. Adherent endotoxins in Ti particles product has been proved to increase the expression of TNF-α, IL-1β and IL-6, which participate in inflammatory response and aseptic prosthetic loosening. We have adopted several steps as indicated in the *Material and Method* section to remove the endotoxins to our greatest extent. In terms of the dosage of Ti particles, from 15 to 30 mg at one time, Ti particles at different volumes were selected in previous researches (Tsutsumi et al., 2008; Shin et al., 2012; Ouyang et al., 2014). However, Margevicius et al. (Margevicius et al., 1994) suggested that different types of wear particles (including UHMWP and metals) accounted for no more than 10% of tissue volume in tissue samples adjacent to failed joint prostheses from patients, which was obvious lower than in animal experiments mentioned above. Even so, we believe that using one-time high-dosage Ti particles implantation might be most reasonable and effective approach in animal models to simulate the particle-induced osteolysis in patient, because such models focus on the local bone erosion rather than accumulation process of wear particles. Therefore, continuous administration of lower concentration particles could be more favorable, but no practical model emerges at present.

Third, the effect of TUS in osteoblast, which also participates in the pathogenesis of aseptic prosthetic loosening (Granchi et al., 2005) was not detected in our research. Also, only the effect of a single TUS concentration was investigated in animals. Whether TUS actually exerts antiosteolysis effect *in vivo* dose-dependently needs further examination. Therefore, the effect of TUS concentration gradient in osteoclast and osteoblast cell models will be studied in the future.

Fourth, we only investigated the involvement of two classical signaling cascades, NF-κB and MAPK pathways *via* Western blot since no information about the involvement of PI3k-Akt, CaMK-CREB, or other pathway in the mechanism of osteoclastogenesis was reported in previous studies. However, these pathways may possibly be responsible for bioactivity of TUS and requires elucidation.

Our work demonstrated that TUS suppresses RANKL-induced NF-κB signaling in osteoclastogenesis from RAW264.7 *via* modulating IκBα degradation without any obvious effect on phosphorylation of IκBα or IKK complex. Comparatively, most previous studies have suggested that TUS suppresses the activation of NF-κB pathway, while the modulated targets vary. Cheon et al. (2018), Choi et al. (2018), Hwang et al. (2018), Kim et al. (2017b), Lee et al. (2016) and Lim et al. (2008) all have reported that TUS blocks NF-κB pathway in exogenous-agent-induced colitis mice, airway epithelial cells, focal cerebral ischemia rats, septic mice, skin inflammation mice, and BV-2 microglial cells by attenuating the nuclear translocation of p65 subunit and the phosphorylation and (or) degradation of IκBα. Among them, Choi et al. (2018) have also observed the alleviation of phosphorylated IKK complex.

Furthermore, we suggested that TUS blocks the p38 cascades without affecting other MAPK signaling pathways, which is partially responsible for inhibited osteoclast differentiation after TUS treatment. However, Hwang et al. (2018) and Kim et al. (2017b) demonstrated that TUS reduces MAPK signaling *via* inhibiting the phosphorylation of not only p38, but also JNK and ERK in focal cerebral ischemia rats and septic mice. In contrast, Hwangbo et al. concluded that TUS cannot modulate MAPK pathways in nuclear factor-erythroid 2-related factor/HO-1 protein production in RAW264.7 using the MAPK-inhibiting assay, as opposed to the above-mentioned hypothesis. To our knowledge, specific dynamical and time-dependent change pattern of MAPK signaling proteins, including JNK, ERK, p38 and their phosphorylated compounds, is essential for the analysis of signaling pathway modulation (Xiao et al., 2015; Ouyang et al., 2019). Therefore the chronological expression of proteins mentioned above in control group (treated with RANKL only) also need quantification. However, the presented Western blot bands in the article of Hwangbo et al. didn't refer any information about the control group or phosphorylated counterparts of these proteins. Therefore, the conclusion drawn from the data that “TUS did not induce the activation of ERK1/2, JNK, and p38MAPKs” (though the author didn't make comment using “inhibition”) is not convincing enough. And we believe that the possible reason for the varied understanding concerning the molecular mechanism of TUS treatment could also be the different agents, cell lines, and animal species used to establish the models in different studies. Moreover, undetected crosstalk between signal pathways can cover up the suppressed upstream molecules and mislead scholars toward pathways where downstream targets are located. Hence, further studies unraveling the entire mechanism network and details of TUS bioactivities are required.

## Conclusion

In summary, for the first time, we discovered that TUS can attenuate osteolysis severity in Ti particle-induced murine calvarium model and verified its antiosteoclastogenesis and antiresorption effect at the cellular level. According to qPCR and Western blotting analyses, these bioactivities may result from the suppression of RANKL-mediated NF-κB and p38-mediated MAPK signaling pathways. Our theory complements the therapeutic effect of TUS in bone-loss osteopathy *via* regulating osteoclasts and broadens the spectrum of antiosteolysis natural compounds, which promises novel potential agents in the treatment of aseptic prosthetic loosening. However, full investigation of TUS targets and more evidence for antiosteolysis clinical adoption are needed.

## Data Availability Statement

All datasets generated for this study are included in the article/supplementary material.

## Ethics Statement

This study was carried out in accordance with the recommendations of guiding principles of Animal Care Committee of Central South University. The protocol was also approved by the Animal Care Committee of Central South University.

## Author Contributions

The study was designed by PD and XH. All procedures were conducted under the quality control of Panel of Laboratory of Osteopathy (DP et al.). XH carried out the literature survey and cell studies, participated in animal studies, and drafted the manuscript. GJ and SL procured the necessary items and participated in cell culture. DY was in charge of animal treatment and sample collection. PD and ZY provided laboratory technique support. ZC and ZL served as advisors for statistical methods and application. DP and XC were responsible for clinical context inquiry. All the authors revised the article critically for intellectual content. All the authors have read and approved the final manuscript.

## Funding

This work was funded by the Science & Technology Department of Hunan Province (Grant number 2017WK2062) and the Natural Science Foundation of Hunan Province for Youths (Grant number 2019JJ50845).

## Conflict of Interest

The authors declare that the research was conducted in the absence of any commercial or financial relationships that could be construed as a potential conflict of interest.

## Abbreviations

BMMs, bone marrow macrophages; BV, bone volume; CCK-8, cell counting kit-8; OD, optical density; CoCrMo, cobalt-chromium-molybdenum; CT, computed tomography; EDTA, ethylene diamine tetraacetic acid; FBS, fetal bovine serum; HO-1, heme oxygenase-1; M-CSF, macrophage colony-stimulating factor; PBS, phosphate buffer solution; PMMA, polymethyl methacrylic; qPCR, quantitative polymerase chain reaction; RANKL, receptor activator of nuclear factor kB ligand; SD, standard deviation; SEM, scanning electron microscope; TBS, tris buffer solution; TRAP, tartrate-resistant acid phosphatase; TUS, tussilagone; TV, total volume.
